# Integrated Analysis of mRNA and microRNA Expression in Corneal Impression Cytology Samples from Patients with *PAX6*-Related Congenital Aniridia

**DOI:** 10.3390/ijms27136088

**Published:** 2026-07-07

**Authors:** Shuailin Li, Tanja Stachon, Fabian Norbert Fries, Mária Csidey, Annamária Náray, Anita Csorba, Ágnes Élő, Berthold Seitz, Zoltán Zsolt Nagy, Erika Maka, Marta Corton, Eszter Jávorszky, Kálmán Tory, Nicole Ludwig, Nóra Szentmáry

**Affiliations:** 1 Dr. Rolf M. Schwiete Center for Limbal Stem Cell and Congenital Aniridia Research, Saarland University, 66424 Homburg, Germany; shuailin.li@uni-saarland.de (S.L.); tanja.stachon@uni-saarland.de (T.S.); fabian.fries@uks.eu (F.N.F.); annamaria.naray@gmail.com (A.N.); 2 Department of Experimental Ophthalmology, Saarland University, 66424 Homburg, Germany; 3 Department of Ophthalmology, Saarland University Medical Center, 66424 Homburg, Germany; berthold.seitz@uks.eu; 4 Department of Ophthalmology, Semmelweis University, 1085 Budapest, Hungary; mcsidey@yahoo.com (M.C.); csorbani@gmail.com (A.C.); eloagi@gmail.com (Á.É.); zoltan.nagy100@gmail.com (Z.Z.N.); dr.maka.erika@gmail.com (E.M.); 5 Department of Genetics and Genomics, Instituto de Investigación Sanitaria-Fundación Jiménez Díaz University Hospital, Universidad Autónoma de Madrid (IIS-FJD, UAM), 28040 Madrid, Spain; marta.corton@gmail.com; 6 Center for Biomedical Network Research on Rare Diseases (CIBERER), Instituto de Salud Carlos III, 28029 Madrid, Spain; 7 MTA-SE Lendület Nephrogenetic Laboratory, Hungarian Academy of Sciences, 1051 Budapest, Hungary; javorszky.eszter@semmelweis.hu (E.J.); tory.kalman@semmelweis.hu (K.T.); 8 1st Department of Pediatrics, Semmelweis University, 1085 Budapest, Hungary; 9 Department of Human Genetics, Saarland University, 66424 Homburg, Germany; n.ludwig@mx.uni-saarland.de

**Keywords:** congenital aniridia, PAX6, mRNA, microRNA, sequencing

## Abstract

This study aimed to measure mRNA and miRNA expression profile in corneal impression cytology (IC) samples from patients with congenital aniridia (CA) and healthy controls, and to elucidate the key genes and signaling pathways involved in aniridia-associated keratopathy (AAK). Corneal IC samples were collected from 14 patients with CA and 14 healthy controls. RNA sequencing was performed to identify differentially expressed genes (DEGs) and miRNAs. Correlations with age and AAK grade were analyzed, selected miRNAs were validated by RT-qPCR, and Gene Ontology (GO) and Kyoto Encyclopedia of Genes and Genomes (KEGG) analyses were conducted to characterize biological functions and pathways. A total of 695 DEGs and 19 differentially expressed miRNAs were identified. *KRT24* expression was negatively associated with age, whereas *LY6D* expression positively correlated with AAK grade. Several miRNAs were linked to disease severity, including positive correlations for miR-224-5p, miR-224-3p, and miR-452-5p, and negative correlations for miR-204-3p, miR-181b-5p, and miR-181a-5p. RT-qPCR confirmed significant downregulation of miR-204-5p and miR-138-5p in aniridia samples. Functional enrichment analyses showed that DEGs were mainly involved in cell adhesion, extracellular matrix remodeling, inflammatory and immune responses, and neural-related processes. Target genes of dysregulated miRNAs were enriched in transcriptional regulation, cell proliferation, apoptosis, and migration, with significant involvement of PI3K-Akt, AGE-RAGE, and EGFR signaling pathways. Corneal epithelial cells from patients with CA exhibit coordinated mRNA and miRNA dysregulation associated with extracellular matrix disruption, inflammation, and altered signaling pathways. These findings improve understanding of AAK pathogenesis and identify potential biomarkers and therapeutic targets.

## 1. Introduction

Congenital aniridia (CA) is a rare genetic disorder characterized by varying degrees of iris hypoplasia and is frequently accompanied by other ocular abnormalities, including nystagmus, secondary glaucoma, lens anomalies, and optic nerve and macular hypoplasia, which can severely compromise visual function. Epidemiological studies indicate that the prevalence of CA ranges from approximately 1:40,000 to 1:100,000 [1,2,3]. The paired box gene 6 (*PAX6*), a key transcription factor involved in ocular development, plays a central role in the pathogenesis of these ocular alterations through haploinsufficiency, which is present in approximately 90% of CA cases [1,4]. Aniridia-associated keratopathy (AAK) represents one of the most severe ocular complications of CA and may ultimately lead to profound deterioration of visual function and a markedly reduced quality of life [5]. AAK is characterized by progressive corneal conjunctivalization and neovascularization, resulting in loss of corneal transparency. Although the exact pathogenic mechanisms underlying AAK remain incompletely understood, limbal epithelial stem cell deficiency is considered a major contributing factor. Increasing evidence also indicates that chronic inflammation, extracellular matrix remodeling, abnormal epithelial differentiation, and corneal neovascularization contribute to disease progression [6,7,8,9,10].

Messenger RNA (mRNA) is essential for cellular function, as it conveys genetic information from DNA to the cytoplasm and directs protein synthesis [11]. Accordingly, analyzing mRNA expression profiles in corneal epithelial cells offers valuable insight into gene-level alterations associated with AAK. Previous studies have demonstrated significant dysregulation of multiple signaling pathways in AAK corneas, including retinoic acid, Notch1, sonic hedgehog (SHH), mTOR, and Wnt/β-catenin signaling [12,13]. High-throughput RNA sequencing enables comprehensive and unbiased profiling of coding transcripts and has become an important approach for identifying disease-associated molecular pathways and potential therapeutic targets [14].

MicroRNAs (miRNAs) are small non-coding RNA molecules, typically 20–24 nucleotides in length, that regulate gene expression by binding to target mRNAs and modulating protein synthesis [15,16,17]. miRNAs are involved in numerous biological processes, including cell proliferation, differentiation, apoptosis, and stress responses. Increasing evidence indicates that miRNAs also play important roles in the development and progression of AAK. For example, miR-204-5p has been reported to regulate *PAX6* expression in corneal epithelial cells and to influence angiogenesis-related factors such as *VEGFA* and *ANGPT1* [18]. It also modulates genes involved in transcriptional regulation, cytoskeletal organization, extracellular matrix remodeling, and retinoic acid signaling in limbal epithelial cells [19]. In addition, miR-7 and miR-375 affect cellular function through regulation of *PAX6* [20], while miR-138-5p, upregulated in corneal cells from patients with CA, targets genes including *FOXC1*, *CASP3*, and *CCND1* [21].

Impression cytology (IC) is a minimally invasive sampling technique in which a nitrocellulose or cellulose acetate membrane is gently applied to the ocular surface to obtain superficial epithelial cells from the conjunctival or corneal surface for cytological, molecular, and immunological analyses [22]. In a previous study, conjunctival IC samples from patients with CA revealed significant downregulation of the anti-angiogenic factor miR-204-5p, along with marked dysregulation of additional mRNAs and miRNAs, suggesting that the conjunctiva in aniridia is maintained in a pro-angiogenic and proliferative state [23]. To date, however, no study has specifically analyzed IC samples obtained from the central corneal surface of patients with CA. This is particularly relevant, as AAK primarily affects the cornea, and potential therapeutic strategies are likely to target this tissue [24]. Therefore, profiling mRNA and miRNA expression in corneal IC samples may accurately reflect site-specific pathogenic mechanisms. Furthermore, integrated analysis of mRNA and miRNA expression provides a more comprehensive understanding of the regulatory networks underlying disease pathogenesis than analysis of either transcript type alone. Such an approach facilitates the identification of key molecular pathways, regulatory interactions, and potential biomarkers that may contribute to the development of targeted therapeutic strategies for AAK.

Therefore, the aim of this study was to investigate the molecular mechanisms underlying AAK by analyzing corneal IC samples from patients with CA. High-throughput sequencing was performed to profile mRNA and miRNA expression patterns and to compare them with those of healthy controls. The objectives were to identify differentially expressed mRNAs and miRNAs, construct potential miRNA–mRNA regulatory networks, and elucidate molecular pathways involved in the development and progression of AAK.

## 2. Results

### 2.1. mRNA and miRNA Expression Profile

**Figure 1:**
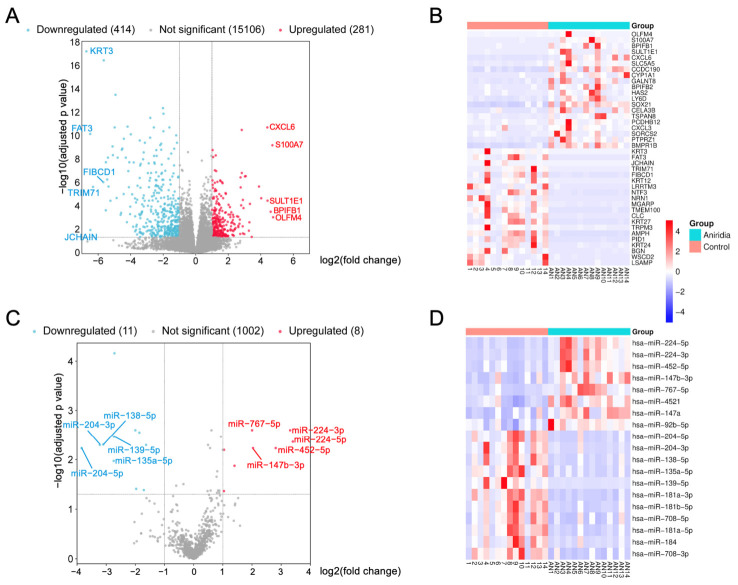
Volcano plot and heatmaps for the differentially expressed mRNAs (**A**,**B**) and miRNAs (**C**,**D**) in corneal IC samples of aniridia subjects versus controls. The volcano plots display the −log10 (adjusted *p*-values) from the *t*-test on the Y-axis and the log2 (fold change) on the X-axis. mRNAs or miRNAs with a log2 (fold change) > 1 and a *p*-value < 0.05 were considered upregulated, and with a log2 (fold change) < −1 and a *p*-value < 0.05 were considered downregulated in corneal IC samples from patients with aniridia (**A**,**C**). Heatmap rows are identified by Entrez gene names or official miRNA names. Intensities are shown by a color range from red (row max) to white (row average) and blue (row minimum) (**B**,**D**).

Of the 20,073 genes analyzed, 15,801 showed expression levels above background in at least 50% of the samples and were therefore included in further analysis. For these genes, a Wald test was performed to compare expression levels between the aniridia and control groups. This analysis identified a total of 695 differentially expressed genes, including 281 upregulated and 414 downregulated genes in superficial corneal cells from patients with aniridia (Figure 1A).

Figure 1B and Table 1 summarize the top 20 upregulated and top 20 downregulated differentially expressed genes (DEGs). Among them, *OLFM4*, *S100A7*, *BPIFB1*, *SULT1E1*, *CXCL6*, and *SLC5A5* were the most strongly upregulated genes, each exhibiting log2 fold change (log2FC) values greater than 4. *KRT3* was the most significantly downregulated gene and showed the largest expression difference between patients with aniridia and healthy controls. Other markedly downregulated genes, including *FAT3*, *JCHAIN*, *TRIM71*, *FIBCD1*, *KRT12*, *LRRTM3*, *NTF3*, *NRN1*, *MGARP*, *TMEM100*, *CLC*, *KRT27*, and *TRPM3* displayed log2FC values less than −5.5.

A similar analytical approach was applied to assess and compare miRNA expression profiles in corneal samples from the two groups. Of the 1103 miRNAs analyzed, 1021 were expressed above background levels in at least 50% of the samples and were included in the analysis. Among these, 19 miRNAs were identified as differentially expressed between the aniridia and control groups. Compared with healthy controls, 8 miRNAs were upregulated and 11 miRNAs were downregulated in corneal samples from patients with aniridia (Figure 1C). The normalized mRNA and miRNA expression matrices, together with the differential expression analysis results, are provided in the Supplementary Data.

Figure 1D and Table 2 present all differentially expressed miRNAs identified between the two groups. Hsa-miR-224-5p, hsa-miR-224-3p, hsa-miR-452-5p, hsa-miR-147b-3p, and hsa-miR-767-5p were the most strongly upregulated miRNAs, each exhibiting log2FC values greater than 2. Hsa-miR-204-5p was the most significantly downregulated miRNA in the corneas of patients with aniridia, with a log2FC of −3.84, and showed the largest expression difference between groups. Other markedly downregulated miRNAs, including hsa-miR-204-3p, hsa-miR-138-5p, hsa-miR-135a-5p, hsa-miR-139-5p, hsa-miR-181a-3p, and hsa-miR-181b-5p, also demonstrated log2FC values below −2.

### 2.2. Correlation Analysis

Among the top 20 upregulated and downregulated mRNAs in aniridia samples, associations between mRNA expression levels and age were generally weak and did not reach statistical significance in either upregulated or downregulated genes, in both aniridia and control samples. With regard to AAK grade, most mRNAs likewise failed to show significant correlations after false discovery rate (FDR) adjustment.

Among the upregulated mRNAs, only *LY6D* displayed a significant positive correlation with AAK grade in aniridia samples (r = 0.809, *p* adj = 0.026), suggesting higher expression with increasing disease severity. *HAS2* showed a similar positive trend (r = 0.728), although this did not remain statistically significant after correction (*p* = 0.078). In the group of downregulated mRNAs, *KRT24* demonstrated a significant negative correlation with age in aniridia samples (r = −0.796, *p* adj = 0.042). However, no significant associations with AAK grade were observed after multiple testing correction. No relevant or statistically significant correlations were detected in control samples (Table 3).

Overall, miRNA expression showed only weak and non-significant associations with age in aniridia samples, regardless of whether the miRNAs were upregulated or downregulated. In contrast, several upregulated miRNAs were strongly and significantly positively correlated with AAK grade in aniridia, including hsa-miR-224-5p (r = 0.756, *p* adj = 0.038), hsa-miR-224-3p (r = 0.735, *p* adj = 0.025), and hsa-miR-452-5p (r = 0.724, *p* adj = 0.019), indicating that their expression levels increase with disease severity.

Conversely, several downregulated miRNAs displayed significant inverse correlations with AAK grade in aniridia, including hsa-miR-204-3p (r = −0.749, *p* adj = 0.029), hsa-miR-181b-5p (r = −0.668, *p* adj = 0.038), and hsa-miR-181a-5p (r = −0.647, *p* adj = 0.044), suggesting that their expression decreases as disease severity increases. No significant correlations were detected in control samples (Table 4).

### 2.3. Target Genes of Dysregulated miRNAs and miRNA-mRNA Binding Site Prediction

**Figure 2:**
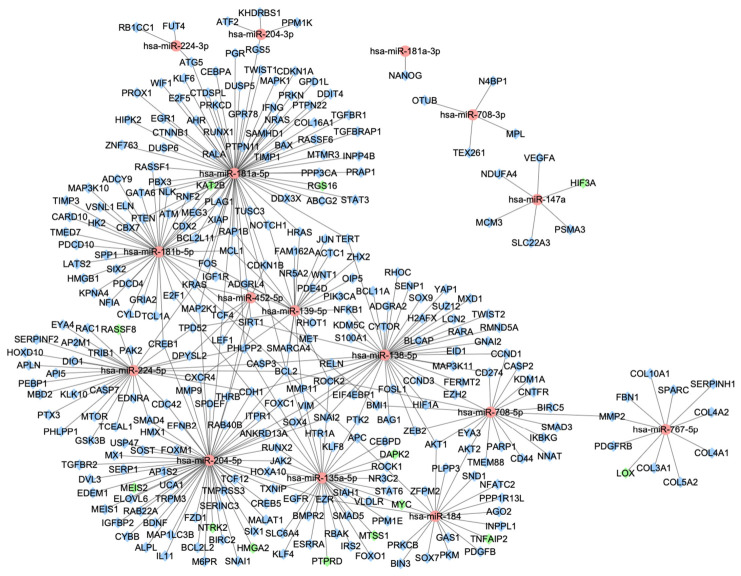
miRNA–mRNA regulatory network in corneal impression cytology (IC) samples from patients with congenital aniridia (CA). Red nodes represent differentially expressed miRNAs. Green nodes indicate experimentally validated target genes that are also differentially expressed in corneal IC samples from patients with CA, whereas blue nodes represent experimentally validated target genes that are not differentially expressed. Edges denote validated regulatory interactions between miRNAs and their target genes. Target genes were identified using the online database miRTarLink 2.0 (https://ccb-compute.cs.uni-saarland.de/mirtargetlink2; accessed on 10 January 2026).

The online tool miRTarLink 2.0 was used to identify target genes regulated by dysregulated miRNAs. Only target genes that had been strongly validated by luciferase reporter assays, immunohistochemistry, microarray analysis, real-time quantitative PCR (RT-qPCR) and Western blotting were included. Using these criteria, validated target genes were identified for 16 of the 19 differentially expressed miRNAs, and the results are summarized in Table 5. Additionally, Figure 2 illustrates the miRNA-mRNA regulatory network constructed from the differentially expressed miRNAs and their experimentally validated target mRNAs in corneal samples from patients with congenital aniridia. A total of 15 dysregulated miRNA-mRNA interaction pairs were identified, in which both the miRNAs and their corresponding target mRNAs were differentially expressed, highlighting potential regulatory interactions associated with AAK pathogenesis. Notably, hsa-miR-181a-5p was associated with the largest number of target genes, with a total of 72 validated targets.

The TargetScanHuman 8.0 platform was used to predict the binding sites of the dysregulated miRNAs and their corresponding target genes that were also differentially expressed in AAK. A total of 15 predicted binding sites were identified across 11 miRNA–mRNA interaction pairs. The predicted binding sites are summarized in Supplementary Table S1.

### 2.4. Protein–Protein Interaction (PPI) Networks and Hub Genes

**Figure 3:**
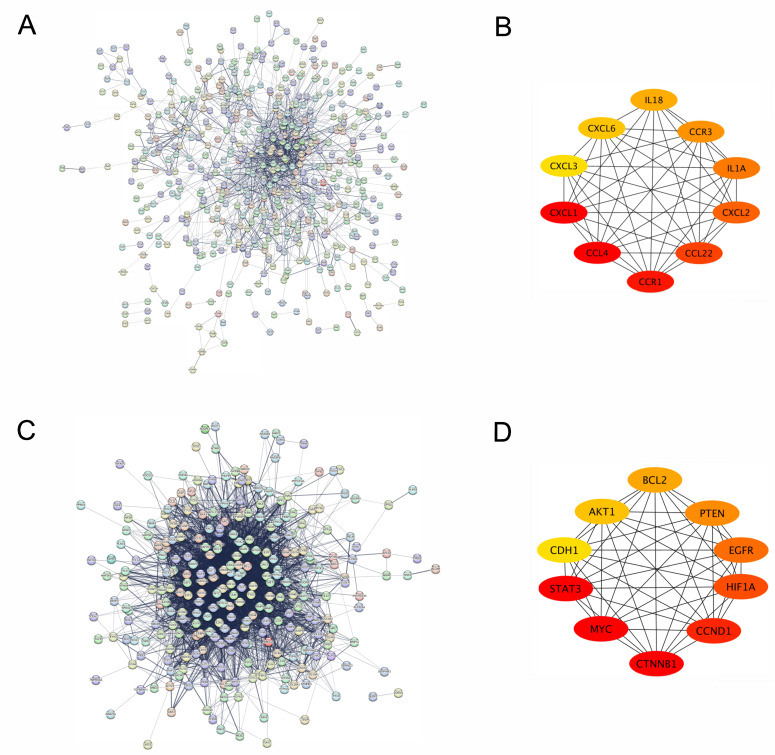
The protein–protein interaction (PPI) network and top 10 hub genes of differently expressed genes (**A**,**B**) and target genes regulated by differently expressed microRNAs (**C**,**D**) in aniridia corneal IC samples. In the PPI network corresponding to DEGs, a total of 686 nodes and 1568 edges were identified (**A**) and in the PPI network of target genes regulated by differentially expressed microRNAs, a total of 296 nodes and 4526 edges were identified (**C**). The color depth refers to the rank of hub genes generated with multiple correlation clustering (MCC) (**B**,**D**).

Figure 3A,C illustrate the PPI networks of DEGs in IC samples of corneas of patients with aniridia and the target genes regulated by differentially expressed microRNAs, respectively.

In the PPI network corresponding to DEGs, a total of 686 nodes and 1568 edges were identified (Figure 3A). The top 10 hub genes were *CXCL1*, *CCL4*, *CCR1*, *CCL22*, *CXCL2*, *IL1A*, *CCR3*, *IL18*, *CXCL6*, and *CXCL13* (Figure 3B).

In the PPI network of target genes regulated by differentially expressed microRNAs, a total of 296 nodes and 4526 edges were identified (Figure 3C). The top 10 hub genes were *STAT3*, *MYC*, *CTNNB1*, *CCND1*, *HIF1A*, *EGFR*, *PTEN*, *BCL2*, *AKT1*, and *CDH1* (Figure 3D).

### 2.5. Gene Ontology (GO) Enrichment Analysis

**Figure 4:**
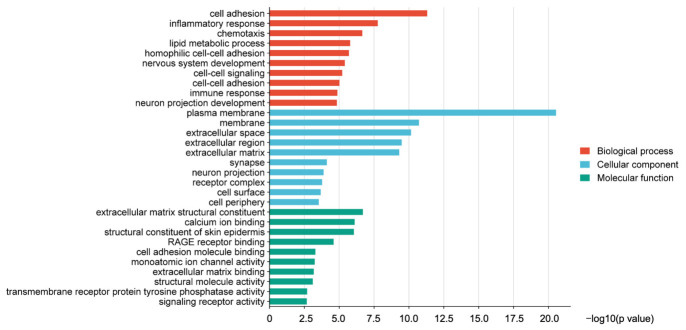
Top 30 Gene Ontology (GO) enrichment terms of differentially expressed mRNAs in aniridia corneal IC samples. The X-axis represents −log10 (*p*-value), and the Y-axis represents the different GO terms.

**Figure 5:**
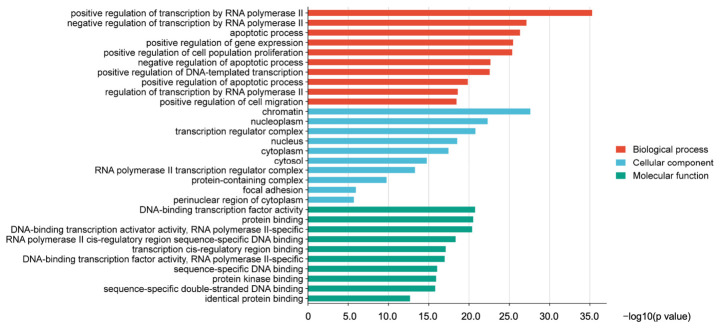
Top 30 Gene Ontology (GO) enrichment terms of target genes regulated by differentially expressed miRNAs in aniridia corneal IC samples. Target genes regulated by differentially expressed miRNAs were identified by using the online tool miRTarLink 2.0 (https://ccb-compute.cs.uni-saarland.de/mirtargetlink2; accessed on 10 January 2026). Only target interactions with strong experimental validation were included, such as those confirmed by luciferase reporter assays, immunohistochemistry, microarray analysis, real-time quantitative PCR (RT-qPCR), and Western blotting. The X-axis represents −log10(*p*-value), and the Y-axis represents the different GO terms.

GO enrichment analysis was performed for both the differentially expressed genes and the target genes regulated by dysregulated miRNAs. Using this standardized functional annotation system, gene functions were categorized into three domains: biological process (BP), cellular component (CC), and molecular function (MF). Under a significance threshold of *p* < 0.05, enriched GO terms were ranked in descending order according to their −log10 *p*-values, and the top 10 terms from each category were presented in Figure 4 and Figure 5.

In the GO enrichment analysis of differentially expressed genes, the most significantly enriched BP terms included cell adhesion, inflammatory response, and chemotaxis. The most enriched CC terms were plasma membrane, membrane, and extracellular space, while the most prominent MF terms included extracellular matrix structural constituent, calcium ion binding, and structural constituent of skin epidermis (Figure 4). The detailed analysis results are provided in Supplementary Table S2.

For the target genes regulated by differentially expressed miRNAs, GO enrichment analysis revealed that the most enriched BP terms were positive and negative regulation of transcription by RNA polymerase II and apoptotic processes. The most enriched CC terms included chromatin, nucleoplasm, and transcription regulator complexes, whereas the most enriched MF terms comprised DNA-binding transcription factor activity, protein binding, and RNA polymerase II–specific DNA-binding transcription activator activity (Figure 5). The detailed analysis results are provided in Supplementary Table S3.

### 2.6. Kyoto Encyclopedia of Genes and Genomes (KEGG) Pathway Analysis

KEGG pathway enrichment analysis was performed for both the differentially expressed genes and the target genes regulated by dysregulated miRNAs. Enriched pathways were ranked in descending order according to their −log10 *p*-values.

**Figure 6:**
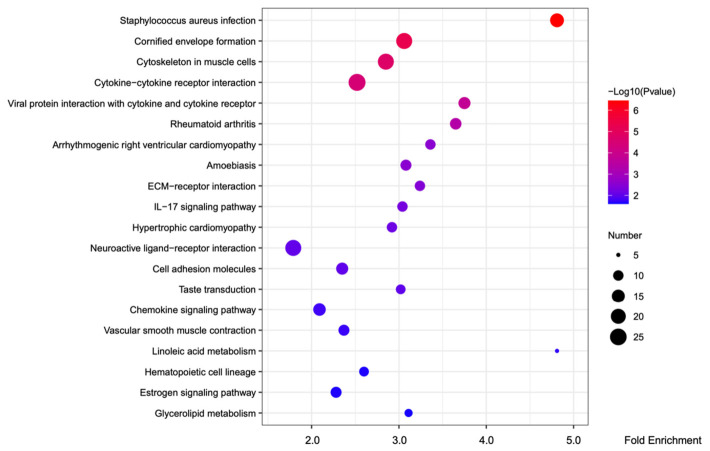
Bubble plot of top 20 Kyoto Encyclopedia of Genes and Genomes (KEGG) enrichment pathway of differentially expressed mRNAs. KEGG pathway enrichment analysis was performed using the set of differentially expressed mRNAs identified in corneal impression cytology samples. In the bubble plot, the size of each circle represents the number of genes associated with the corresponding pathway, while the color of the circle indicates the *p*-value, reflecting the statistical significance of pathway enrichment.

KEGG pathway analysis of differentially expressed genes revealed that the most significantly enriched pathways included *Staphylococcus aureus* infection, cornified envelope formation, cytoskeleton organization in muscle cells, cytokine-cytokine receptor interaction, and viral protein interaction with cytokine and cytokine receptors, among others (Figure 6). The detailed analysis results are provided in Supplementary Table S4.

**Figure 7:**
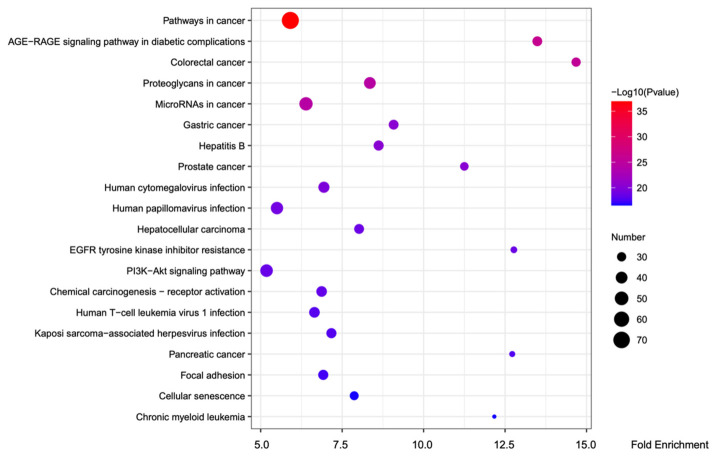
Bubble plot of top 20 Kyoto Encyclopedia of Genes and Genomes (KEGG) enrichment pathway of target genes regulated by differentially expressed miRNAs. Target genes regulated by differentially expressed miRNAs were identified by using the online tool miRTarLink 2.0 (https://ccb-compute.cs.uni-saarland.de/mirtargetlink2; accessed on 10 January 2026). Only target interactions with strong experimental validation were included, such as those confirmed by luciferase reporter assays, immunohistochemistry, microarray analysis, real-time quantitative PCR (RT-qPCR), and Western blotting. The number of genes was represented by the size of the circle, and the *p*-value was represented by the color of the circle.

Similarly, KEGG pathway analysis of target genes regulated by differentially expressed miRNAs demonstrated significant enrichment in pathways related to cancer, the AGE-RAGE signaling pathway in diabetic complications, colorectal cancer, proteoglycans in cancer, and microRNAs in cancer (Figure 7). The detailed analysis results are provided in Supplementary Table S5.

### 2.7. RT-qPCR Validation

**Figure 8:**
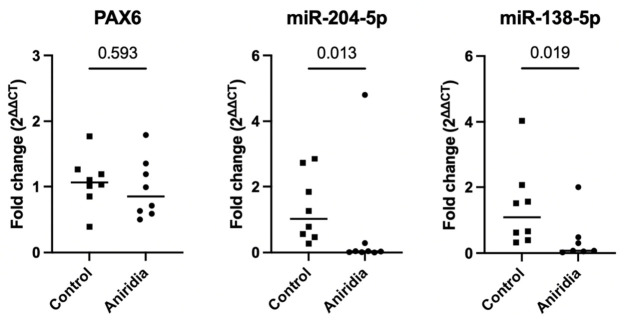
RT-qPCR validation results of PAX6, miR-204-5p, and miR-138-5p expression levels in corneal impression cytology (IC) samples from healthy controls and patients with aniridia (*n* = 8). Expression levels were quantified by RT-qPCR and are presented on a logarithmic scale (log_2_). Statistical comparisons between aniridia and healthy control samples were performed using the Mann–Whitney test. The expression levels of miR-204-5p and miR-138-5p were significantly reduced in aniridia IC samples compared with healthy controls (*p* = 0.013 and *p* = 0.019, respectively), whereas PAX6 expression showed no significant difference (*p* = 0.593). Significant *p*-values (<0.05) are indicated in the plots.

Among the differentially expressed miRNAs, we selected miR-204-5p and miR-138-5p, two miRNAs that have previously been demonstrated to play important roles in AAK for RT-qPCR validation. Compared with healthy controls, the expression level of miR-204-5p in corneal aniridia IC samples was significantly decreased (*p* = 0.013). Similarly, the expression level of miR-138-5p in corneal aniridia IC samples was also significantly reduced (*p* = 0.019). In addition, we also performed RT-qPCR validation for *PAX6*. The RNA sequencing results showed that *PAX6* was not downregulated (log2FC = 0.08784, *p* = 0.651) and the RT-qPCR results also indicated that there was no statistically significant difference in *PAX6* expression levels between aniridia samples and control samples (*p* = 0.593) (Figure 8).

## 3. Discussion

In this study, we systematically profiled and compared mRNA and miRNA expression patterns in corneal IC samples of patients with CA and healthy controls and performed GO and KEGG pathway enrichment analyses on both DEGs and the target genes regulated by differentially expressed miRNAs. In contrast to previous transcriptomic studies that analyzed conjunctival IC specimens and primary limbal epithelial and stromal cell cultures from patients with CA [21,23], the present investigation focused specifically on superficial cells collected from the central and paracentral corneal surface. Because these samples were mainly obtained from the most superficial epithelial layer, they were expected to represent a more differentiated cell population. This approach allows for a more direct assessment of cornea-specific alterations at the mRNA and miRNA levels in patients with AAK.

Among the 695 identified DEGs, 414 genes (59.6%) were downregulated, with *KRT3* exhibiting the largest expression difference compared with the control group. In addition, *KRT12*, *KRT24*, and *KRT27* were also markedly downregulated. Notably, *KRT3* and *KRT12* are recognized as specific markers of corneal epithelial maturation and differentiation, and their reduced expression indicates impaired corneal epithelial differentiation, increased epithelial fragility, and epithelial conjunctivalization in patients with aniridia [25,26,27]. These findings are highly consistent with the role of *PAX6* as a key regulator of ocular surface development, and the decreased expression of *KRT3* and *KRT12* may represent downstream consequences of *PAX6* dysfunction [28,29]. Furthermore, several genes associated with cell adhesion, including *FAT3*, *OLFM4*, *LRRTM3*, *PCDHB12*, and *TSPAN8* [30,31], are also regulated by *PAX6* [32]. Dysregulation of these genes suggests compromised corneal stability in AAK, rendering the cornea more susceptible to mechanical injury [33]. In addition, altered expression of genes closely related to neuronal development, regulation, and sensory function, such as *NTF3*, *NRN1*, *LSAMP*, *SORCS2*, *PTPRZ1*, and *TRPM3*, together with numerous inflammation-related genes, including *S100A7*, *CXCL3*, *CXCL6*, *JCHAIN*, *FIBCD1*, and *CLC*, may help explain clinical features observed in patients with AAK, such as reduced corneal innervation and increased inflammatory cell infiltration [7,34].

Among the 19 differentially expressed miRNAs identified in this study, hsa-miR-204-5p exhibited the largest expression difference between corneal IC samples from patients with aniridia and healthy controls and was markedly downregulated in aniridia. miR-204-5p has been reported to act as a regulatory factor of *PAX6* [18] and is involved in corneal epithelial cell differentiation, migration, and inflammatory responses [19]. Using the online tool miRTarLink 2.0, we identified 60 strongly validated target genes of miR-204-5p. Functionally, these targets are involved in corneal epithelial differentiation, cell adhesion and migration, apoptosis, inflammation, and immune responses, as well as in key signaling pathways, including Wnt/β-catenin, TGF-β, and MAPK. Dysregulation of these targets may collectively contribute to impaired epithelial differentiation, enhanced cell migration, persistent inflammatory activation, and neural dysfunction observed in AAK [23,35,36]. Analysis of conjunctival IC samples from patients with aniridia by Latta et al. yielded comparable results, demonstrating a 26.79-fold downregulation of miR-204-5p expression [23]. As miR-204-5p functions as an inhibitor of neovascularization, its reduced expression may contribute to the development of corneal neovascularization in patients with AAK [18,23].

In addition to hsa-miR-204-5p, other dysregulated miRNAs, including hsa-miR-224-5p and hsa-miR-138-5p, may also influence a broad range of cellular functions through regulation of their target genes and play important roles in the development and progression of AAK [21,37]. Therefore, miRTarLink 2.0 was used to identify strongly validated target genes regulated by all upregulated and downregulated miRNAs. A total of 31 genes have been identified as strongly regulated targets of miR-224-5p, many of which are implicated in the control of cell proliferation, differentiation, and apoptosis [38,39,40]. In the present study, miR-224-5p was markedly upregulated in corneal IC samples from patients with aniridia, showing an approximately 10.56-fold increase. In comparison, conjunctival cells from aniridia patients exhibited a more moderate upregulation of approximately 2.44-fold [23], indicating a more pronounced dysregulation in the cornea. Previous studies further suggest that miR-224-5p can enhance cellular proliferation and migration [41,42]. Accordingly, its elevated expression may contribute to the hyperproliferative and migratory phenotype observed in AAK corneal epithelium, potentially promoting disease progression.

In addition, we analyzed the correlations between differentially expressed mRNAs and miRNAs and parameters such as age and AAK grade. Among the differentially expressed mRNAs, *KRT24* expression showed a significant negative correlation with patient age in aniridia samples, whereas no such association was observed in healthy controls. *KRT24*, a member of the keratin family, has been identified as a potential marker of cellular differentiation and an antiproliferative factor [43]. The age-related decline in *KRT24* expression may reflect progressive alterations in corneal epithelial homeostasis during disease progression in patients with aniridia [24]. In contrast, the absence of this correlation in healthy samples suggests that this age-related change may be associated with the pathological microenvironment of aniridia. Furthermore, *LY6D* expression was significantly positively correlated with AAK grade, suggesting a potential association with disease severity. Previous studies have shown that *LY6D* is involved in epithelial differentiation and immune-related processes [44,45]. Therefore, the increased expression of *LY6D* with higher AAK grades may reflect enhanced epithelial remodeling or inflammatory responses during the progression of AAK [46].

Regarding miRNAs, although no significant correlations were observed between miRNA expression levels and age, several miRNAs were significantly associated with AAK grade. Specifically, the expression levels of hsa-miR-224-5p, hsa-miR-224-3p, and hsa-miR-452-5p were positively correlated with AAK grade, whereas hsa-miR-204-3p, hsa-miR-181b-5p, and hsa-miR-181a-5p showed negative correlations with AAK grade. These findings suggest that dysregulated miRNAs may contribute to the progression of AAK through the regulation of target genes involved in disease-related biological processes.

Due to the limited amount of total RNA, we selected miR-204-5p and miR-138-5p, two miRNAs that have been demonstrated to play important roles in the pathogenesis of AAK [18,47], and validated their expression levels using RT-qPCR. The RT-qPCR results showed that the expression levels of miR-204-5p and miR-138-5p were markedly reduced in IC samples of corneas of patients with aniridia, which was consistent with the results of our sequencing analysis. Furthermore, we also performed RT-qPCR validation for *PAX6*. Although *PAX6* is a key gene implicated in aniridia, our results showed no significant change in its mRNA expression levels in AAK IC samples. This may be explained by the fact that *PAX6*-associated pathogenesis is often due to haploinsufficiency or functional impairment rather than transcriptional downregulation. In addition, post-transcriptional regulation, protein dysfunction, and tissue heterogeneity may contribute to the discrepancy between gene expression and phenotypic manifestation [48].

We performed PPI network analysis and identified the top 10 hub genes for both DEGs and the target genes regulated by differentially expressed miRNAs, with the aim of identifying genes that may play critical roles in the development and progression of AAK. The results showed that all of the top 10 hub genes among the DEGs in aniridia corneas were associated with inflammation, further indicating that inflammation-related pathways are markedly activated in AAK [49]. In contrast, the results of PPI network analysis and hub gene selection for the target genes regulated by differentially expressed miRNAs were more diverse. These hub genes are mainly involved in signaling pathways such as PI3K/Akt, JAK–STAT, and Wnt/β-catenin, and participate in processes including inflammatory responses, cell cycle regulation, cell proliferation, and cell adhesion [21,23].

Thereafter, target genes regulated by other differently expressed miRNAs were subsequently subjected to GO and KEGG pathway enrichment analyses to further characterize their functional relevance. GO enrichment analysis of DEGs revealed significant enrichment of BP terms related to cell adhesion, cell–cell adhesion, and homophilic cell–cell adhesion, indicating substantial disruption of intercellular junctions and structural stability in the corneal epithelium. This finding is consistent with the marked downregulation of corneal epithelial differentiation markers, such as *KRT3* and *KRT12*, observed in this study, and supports the pathological features of impaired epithelial differentiation and increased epithelial fragility in AAK [50]. In addition, multiple GO terms associated with inflammatory response, immune response, and chemotaxis were significantly enriched, suggesting persistent inflammatory activation in the corneas of patients with aniridia, as also described previously [7,51,52]. This observation aligns with the dysregulated expression of numerous inflammation- and chemotaxis-related genes among the DEGs, including *CXCL3*, *CXCL6*, and *S100A7*, and provides a molecular basis for the commonly observed inflammatory cell infiltration and corneal neovascularization in AAK [7]. Notably, GO terms related to nervous system development and neuron projection development were also significantly enriched, indicating potential impairment of corneal nerve development and maintenance. This finding is consistent with clinical observations of reduced corneal nerve density and decreased corneal sensitivity in patients with AAK [51,53], and is further supported by the dysregulated expression of neuron-associated genes identified in this study, such as *NTF3* and *NRN1*. At the CC and MF levels, DEGs were predominantly localized to the plasma membrane, extracellular space, and extracellular matrix and were enriched in functions related to structural molecule activity, cell adhesion molecule binding, and calcium ion binding. These results further indicate that both the structural integrity and functional properties of the corneal epithelium are compromised in AAK. Collectively, these GO enrichment findings suggest that epithelial structural instability, persistent inflammatory activation, and neural dysfunction jointly contribute to the molecular basis of AAK pathogenesis [54]. In contrast, GO enrichment analysis of the target genes regulated by differentially expressed miRNAs predominantly highlighted upstream regulatory mechanisms related to transcriptional control and cell fate determination (Figure 5).

At the BP level, enriched terms included RNA polymerase II–mediated transcription, regulation of DNA-templated transcription, general gene expression control, as well as regulation of cell proliferation, migration, and apoptosis. These findings suggest that dysregulated miRNAs may exert their effects primarily by modulating key transcriptional programs, thereby influencing fundamental corneal epithelial processes that contribute to AAK progression [24,55] (Figure 5).

At the CC level, significant enrichment was observed in chromatin, nucleoplasm, nucleus, transcriptional regulatory complexes, and cytoplasm, further supporting the notion that these target genes are closely linked to transcriptional regulation and intracellular gene expression machinery. At the molecular function level, the target genes were mainly associated with DNA-binding transcription factor activity and transcriptional activator functions. Collectively, these results indicate that altered miRNA expression may disrupt upstream transcription factors and their regulatory networks, ultimately impairing corneal epithelial proliferation, migration, and apoptosis. Dysregulation of the miRNA-mRNA interaction network therefore likely represents a key molecular mechanism underlying the development and progression of AAK (Figure 5).

KEGG pathway analyses of both the DEGs and the target genes regulated by differentially expressed miRNAs further supported our hypotheses from complementary perspectives. The KEGG enrichment results encompass six major functional categories: metabolism, genetic information processing, environmental information processing, cellular processes, organismal systems, and human diseases [55]. Based on the KEGG pathway analysis of DEGs, genes differentially expressed in the corneal epithelium of patients with CA were primarily enriched in pathways related to inflammatory and immune responses, cell adhesion and extracellular matrix remodeling, and neural and sensory signaling. These findings are highly consistent with the known pathological features of AAK [56,57]. Similarly, KEGG analysis of target genes regulated by dysregulated miRNAs revealed enrichment in pathways associated with key cellular signal transduction, cell adhesion and ECM remodeling, cell proliferation and senescence, and inflammatory and immune responses. Among the most significantly enriched pathways, the PI3K-Akt signaling pathway, AGE-RAGE signaling pathway, and EGFR-related pathways were prominently represented. These pathways play central roles in regulating cell proliferation, survival, stress responses, and cell migration [18,58,59]. Broad miRNA-mediated regulation of critical components within these signaling cascades may therefore contribute to impaired corneal epithelial differentiation, aberrant cell migration, and dysregulated repair processes.

In addition, KEGG enrichment analysis identified numerous pathways annotated with other diseases. It should be emphasized that the enrichment of these pathways does not imply a direct disease association between aniridia and these conditions. Rather, it likely reflects substantial overlap between key transcription factors or core signaling molecules involved in these pathways and genes relevant to aniridia. These genes are broadly involved in fundamental biological regulation and represent shared molecular mechanisms across multiple diseases. Notably, multiple cancer-associated signaling pathways were significantly enriched in our dataset. This observation may relate to the central role of *PAX6* as a master transcription factor, whose dysregulation has been implicated in the development and progression of various malignancies [60]. Depending on the cellular context, *PAX6* may function either as an oncogene or as a tumor suppressor [61,62,63]. Although direct clinical evidence linking CA to increased cancer incidence remains limited, our findings point to a potential molecular association that merits careful consideration and further investigation.

We have previously analyzed the mRNA expression profiles of limbal epithelial cells (AN-LECs) and limbal stromal cells (AN-LSCs) in patients with CA [49], thereby investigating gene expression changes in AAK at the level of limbal stem cells. In contrast, the present study focuses on corneal IC samples from patients with aniridia, which represent the mature phenotype of AAK. Although all three cell types examined across the two studies exhibited significant alterations in inflammation-related genes and signaling pathways, notable differences were observed in other functional gene categories and pathways. DEGs in aniridia corneal IC samples were mainly enriched in extracellular matrix remodeling, cell adhesion, cell proliferation, and cellular senescence, suggesting structural abnormalities of the corneal epithelium and impairment of barrier function. In AN-LECs, the predominant changes were observed in immune- and interferon-related pathways, indicating intrinsic dysfunction of epithelial cells. In contrast, AN-LSCs were mainly characterized by dysregulation of metabolic and mitochondrial pathways, suggesting that abnormalities in the stromal microenvironment contribute to impaired limbal stem cell function in AAK [49].

By analyzing both DEGs and the target genes regulated by differentially expressed miRNAs, we were able to elucidate the molecular mechanisms underlying AAK from complementary perspectives. Integration of GO and KEGG enrichment analyses at these two levels suggests that disruption of the miRNA regulatory network represents an upstream driving force in AAK pathogenesis. Aberrant miRNA expression initially affects key regulatory processes, including transcriptional control, cell proliferation, migration, and apoptosis, which subsequently leads to impaired corneal epithelial differentiation and phenotypic instability.

In contrast, DEGs were predominantly enriched in pathways related to cytokine and chemokine signaling, ECM-receptor interactions, cytoskeletal organization and cell adhesion, as well as neural and sensory signaling. These findings reflect downstream pathological changes, such as reduced cell adhesion capacity, abnormal extracellular matrix organization, impaired corneal innervation, and persistent inflammatory activation. Collectively, these results provide systematic molecular evidence for the pathogenesis of AAK and highlight the critical role of the miRNA-mRNA regulatory network in the development and progression of this disease.

Nevertheless, this study has several limitations. First, the relatively small sample size may have limited the statistical power to detect associations between gene/miRNA expression and clinical parameters. Although several mRNAs and miRNAs showed nominally significant correlations with age or AAK grade, only a limited number of these associations remained significant after correction for multiple testing. These findings suggest that the relationships between molecular expression profiles and clinical characteristics are complex and should be interpreted with caution. Further validation in larger, independent patient cohorts is warranted. Second, the biological functions and regulatory mechanisms of the identified dysregulated genes and miRNAs were primarily inferred from transcriptomic profiling, functional enrichment analyses, and experimentally validated miRNA–mRNA interactions reported in the literature and public databases. Therefore, direct functional studies are still required to validate their specific biological roles and elucidate the underlying molecular mechanisms involved in the pathogenesis of AAK. In addition, owing to the limited quantity of RNA obtained from corneal impression cytology samples, we were unable to perform RT-qPCR validation of additional key genes and dysregulated miRNAs. Finally, because this study was conducted in a relatively small cohort using a specific patient population and a single sampling method, the generalizability of our findings to broader populations with different disease stages, genetic backgrounds, and demographic characteristics remains to be established. Future multicenter studies including larger and more diverse cohorts, together with comprehensive functional investigations, are warranted to validate our findings and further elucidate the molecular mechanisms underlying AAK.

## 4. Materials and Methods

### 4.1. Ethical Considerations

The study was conducted in accordance with the principles of the Declaration of Helsinki. Written informed consent was obtained from all participants or their legal guardians, and the study protocol was approved by the Ethics Committee of Semmelweis University, Budapest, Hungary (No. 80/2020) and by the Ethics Committee of the Medical Association of Saarland, Germany (No. 110/17).

### 4.2. IC Sample Collection

**Figure 9:**
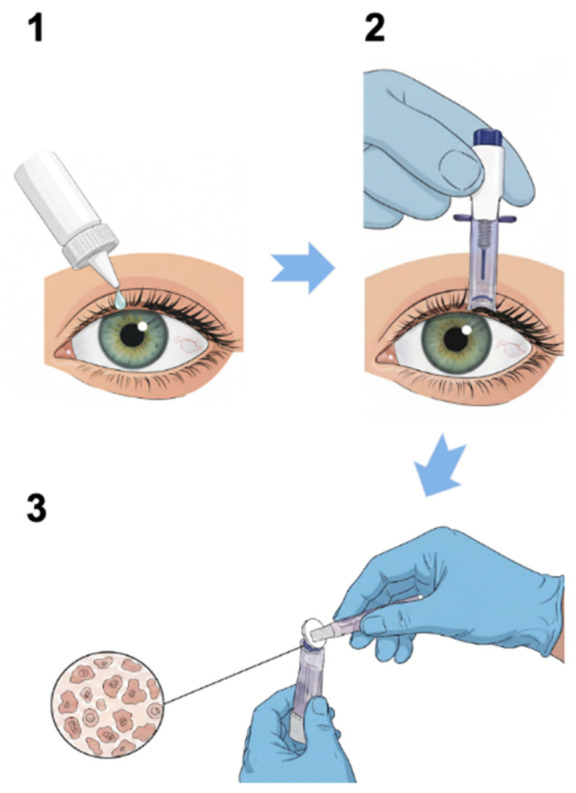
Procedure for collecting corneal impression cytology (IC) samples. Topical anesthesia was achieved by instilling 0.5% proparacaine hydrochloride eye drops into the conjunctival sac (1). Corneal IC samples were subsequently obtained using the EYEPRIM™ device (OPIA Technologies, Paris, France) (2). The applicator was carefully positioned on the corneal surface, aligned from the central cornea toward the inferior limbus. Light, steady pressure was applied for about 2–3 s, after which the device was gently lifted perpendicular to the surface to collect the sample. The membrane containing corneal surface cells was immediately transferred into lysis buffer provided with the DNA/RNA/Protein Purification Kit Micro (Norgen Biotek Corp., Thorold, ON, Canada) (3) and stored at −80 °C until RNA isolation.

IC samples from patients with CA were collected at the Department of Ophthalmology, Semmelweis University, Budapest, Hungary, while samples from healthy controls were obtained at the Department of Ophthalmology, Saarland University Medical Center, Homburg/Saar, Germany. The IC sampling procedure is illustrated in Figure 9. First, 0.4% oxybuprokain-hidroklorid eye drops were instilled into the conjunctival sac to achieve topical anesthesia. Samples were then collected using the EYEPRIM™ device (OPIA Technologies, Paris, France), which has a sampling area of approximately 69 mm^2^. The applicator head was gently applied to the corneal surface, with its long axis oriented from the central cornea toward the inferior limbus. Gentle pressure was maintained for approximately 2–3 s, after which the device was lifted vertically to obtain the corneal IC sample. The collected membrane was immediately transferred into lysis buffer provided by the DNA/RNA/Protein Purification Kit Micro (Norgen Biotek Corp., Thorold, ON, Canada) and stored at −80 °C until RNA extraction.

For the present study, IC specimens were collected from 14 patients with CA (mean age: 25.6 ± 15.6 years; range: 11–57 years; 7 males and 7 females) and 14 healthy controls (mean age: 29.2 ± 19.6 years; range: 2–60 years; 6 males and 8 females). The demographic and clinical characteristics of the study population are summarized in Table 6.

### 4.3. RNA Isolation and Quality Control

Total RNA, including miRNA, was isolated from all IC specimens using the DNA/RNA/Protein Purification Kit Micro (Norgen Biotek Corp., Thorold, ON, Canada) according to the manufacturer’s instructions. Following purification, RNA was eluted in 30 μL of nuclease-free water. For quality control, total RNA concentration was determined using a UV/VIS spectrophotometer (Analytik Jena AG, Jena, Germany).

### 4.4. Whole-Transcriptome and miRNome Sequencing and Data Analysis

Global transcriptome analysis was carried out using IC-derived RNA samples from 14 patients with CA and 14 healthy controls. The samples were processed in separate experimental batches. RNA sequencing libraries were prepared with the MGIEasy rRNA Depletion Kit together with the MGIEasy Universal Library Prep Set (MGI Tech, Shenzhen, China) according to the manufacturer’s protocol. Sequencing was performed at the Sequencing Unit of the Core Facility for Molecular Single Cell and Particle Analysis, Saarland University, using a DNBSEQ-G400RS platform with 100 bp paired-end reads.

RNA-Seq data processing and quantification were carried out using the mRNA workflow implemented in snakePipes [64]. Sequencing reads were aligned to the human reference genome (GRCh38) at the gene level using STAR [65], and gene-level read counts were obtained with FeatureCounts [66]. Quality control was performed with FastQC, and results were aggregated using multiQC to ensure overall sequencing quality and integrity [67]. Raw count data were subjected to variance-stabilizing transformation (VST) using DESeq2 (version 1.46.0) [32], and the transformed data were used for clustering analyses.

For microRNA analysis, sequencing libraries were generated using the MGIEasy Small RNA Library Prep Kit (MGI Tech, Shenzhen, China) following the manufacturer’s instructions. Sequencing was performed on the same DNBSEQ-G400RS platform using a 50 bp single-end read configuration. The resulting FASTQ files were analyzed with the miRmaster 2.0 pipeline [68], which includes adapter trimming, read collapsing, and miRNA quantification based on miRBase v22.1 [69]. To enable cross-sample comparisons, miRNA expression levels were normalized to reads per million mapped miRNAs (RPMMM).

Differential gene and miRNA expression analyses were performed using the DESeq2 package. Raw count data were modeled using a negative binomial distribution, and differential expression was assessed using the Wald test. *p*-values were adjusted for multiple testing using the Benjamini–Hochberg false discovery rate (FDR) procedure. Genes and miRNAs with an absolute fold change > 2 (or < 0.5) and an adjusted *p*-value < 0.05 were considered differentially expressed.

### 4.5. Correlation Analysis

To assess whether the expression levels of differentially expressed mRNAs and miRNAs were associated with age and AAK grade, Spearman correlation analysis was performed. Prior to analysis, data distribution was evaluated using the Shapiro–Wilk test. *p*-values were adjusted for multiple testing using the Benjamini–Hochberg method to control the FDR, with a significance threshold set at 0.05.

### 4.6. Prediction of miRNA-mRNA Regulatory Relationships and Binding Site Prediction

We used the online tool miRTarLink 2.0 (https://ccb-compute.cs.uni-saarland.de/mirtargetlink2; accessed on 10 January 2026) to identify target genes regulated by dysregulated miRNAs [70]. Only experimentally validated target interactions were included, based on evidence from luciferase reporter assays, immunohistochemistry, microarray analysis, RT-qPCR, and Western blotting.

The TargetScanHuman 8.0 platform (https://www.targetscan.org/vert_80/; accessed on 26 June 2026) was used to predict miRNA–mRNA binding sites. Based on the miRNA–mRNA regulatory network generated using miRTarLink 2.0, binding site prediction was performed for the 15 miRNA–mRNA interaction pairs in which both the miRNAs and their corresponding target genes were differentially expressed in corneal IC samples from patients with CA.

### 4.7. PPI Network and Hub Gene Identification

To identify functional relationships, PPI networks of differentially expressed mRNAs and the target genes of differentially expressed microRNAs in aniridia corneal samples were constructed using the STRING database (https://string-db.org; accessed on 10 January 2026). The minimum required interaction score was set to 0.4.

To further identify genes with high connectivity within the PPI network, those likely to play critical roles in network regulation, we performed maximal clique centrality (MCC) analysis using the cytoHubba plugin in Cytoscape (version 3.10.4) and selected the top 10 hub genes.

### 4.8. KEGG Pathway Enrichment and GO Analysis

To uncover the functions of DEGs and the target genes regulated by differentially expressed miRNAs, and to further investigate the pathways among co-regulated genes, we performed GO analysis, including BP, MF, and CC, as well as KEGG pathway analysis using the DAVID Bioinformatics online platform (https://davidbioinformatics.nih.gov; accessed on 10 January 2026).

### 4.9. RT-qPCR Validation

For mRNA validation, 500 ng of total RNA was reverse-transcribed into complementary DNA (cDNA) using the OneTaq RT-PCR Kit (New England Biolabs GmbH, Frankfurt am Main, Germany). RT-qPCR was subsequently performed on a QuantStudio 5 Real-Time PCR System (Applied Biosystems, Waltham, MA, USA) using ACEq SYBR Green Master Mix (Vazyme Biotech, Nanjing, China). Glucuronidase Beta (GUSB) and TATA-box binding protein (TBP) were selected as internal reference genes for normalization.

For miRNA validation, cDNA synthesis was conducted using the miRCURY LNA miRNA RT Kit, followed by duplicate qPCR reactions with the miRCURY LNA miRNA SYBR Green PCR Kit (Qiagen GmbH, Hilden, Germany), according to the manufacturer’s instructions. miR-103a-3p and snRNA RNU6 were used as endogenous controls for normalization.

Relative expression levels were calculated using the ΔΔCt method. Normality was assessed using the Shapiro–Wilk test and then Mann–Whitney test was used to compare the expression level of mRNA and miRNAs in healthy control and aniridia IC corneal samples.

## 5. Conclusions

In this study, we systematically profiled mRNA and miRNA expression patterns in corneal IC samples of patients with CA and healthy controls and identified 695 DEGs and 19 differentially expressed miRNAs. Several mRNAs and miRNAs were significantly associated with age or AAK severity. However, after correction for multiple testing, only a limited number of these associations remained significant, indicating that their potential involvement in disease progression should be interpreted with caution and requires further validation in larger, independent cohorts. Functional enrichment analyses of the DEGs and miRNA target genes using GO and KEGG pathways suggest that dysregulation of the miRNA–mRNA regulatory network contributes to the pathogenesis of AAK. Aberrant miRNA expression may disrupt gene regulatory programs involved in corneal epithelial cell proliferation, migration, differentiation, and apoptosis. These molecular alterations may, in turn, contribute to the characteristic clinical features of AAK, including impaired epithelial integrity, increased corneal fragility, reduced corneal nerve density and sensitivity, and persistent activation of inflammatory and immune-related pathways.

## Figures and Tables

**Table 1:** Top 20 upregulated and downregulated differentially expressed genes (DEGs) in corneas of patients with congenital aniridia.

Upregulated			Downregulated		
Gene	log2FC	Adjusted *p*-Value	Gene	log2FC	Adjusted *p*-Value
*OLFM4*	4.76053	0.00097	*KRT3*	−6.72164	6.12 × 10^−18^
*S100A7*	4.71830	6.67 × 10^−10^	*FAT3*	−6.48692	7.16 × 10^−11^
*BPIFB1*	4.61029	0.00033	*JCHAIN*	−6.48013	0.01193
*SULT1E1*	4.41796	3.67 × 10^−5^	*TRIM71*	−6.31313	2.46 × 10^−6^
*CXCL6*	4.40499	1.99 × 10^−11^	*FIBCD1*	−5.68106	9.62 × 10^−7^
*SLC5A5*	4.02887	2.27 × 10^−5^	*KRT12*	−5.65457	3.61 × 10^−17^
*CCDC190*	3.89206	2.27 × 10^−6^	*LRRTM3*	−5.52953	0.00025
*CYP1A1*	3.44465	0.04616	*NTF3*	−5.52431	1.90 × 10^−8^
*GALNT8*	3.33706	6.94 × 10^−5^	*NRN1*	−5.44293	5.70 × 10^−7^
*BPIFB2*	3.30247	0.00260	*MGARP*	−5.39803	7.65 × 10^−9^
*HAS2*	3.27081	0.01365	*TMEM100*	−5.36246	2.27 × 10^−6^
*LY6D*	3.07574	2.92 × 10^−7^	*CLC*	−5.24381	3.73 × 10^−5^
*SOX21*	3.02740	3.11 × 10^−7^	*KRT27*	−5.11414	8.43 × 10^−10^
*CELA3B*	2.99018	0.02642	*TRPM3*	−5.09350	4.70 × 10^−9^
*TSPAN8*	2.93999	0.00016	*AMPH*	−4.98289	1.99 × 10^−11^
*PCDHB12*	2.90363	0.02331	*PID1*	−4.93185	3.23 × 10^−14^
*CXCL3*	2.90193	3.17 × 10^−5^	*KRT24*	−4.86191	2.18 × 10^−5^
*SORCS2*	2.88825	0.00580	*BGN*	−4.80152	7.65 × 10^−9^
*PTPRZ1*	2.83504	1.57 × 10^−7^	*WSCD2*	−4.61720	0.00059
*BMPR1B*	2.83231	3.25 × 10^−11^	*LSAMP*	−4.58350	0.00041

**Table 2:** Upregulated and downregulated microRNAs in corneas of patients with congenital aniridia.

Upregulated			Downregulated		
Gene	log2FC	Adjusted *p*-Value	Gene	log2FC	Adjusted *p*-Value
*hsa-miR-224-5p*	3.40072	0.00433	*hsa-miR-204-5p*	−3.84417	0.00586
*hsa-miR-224-3p*	3.30741	0.00252	*hsa-miR-204-3p*	−3.21919	0.00505
*hsa-miR-452-5p*	2.81266	0.00586	*hsa-miR-138-5p*	−3.10140	0.00496
*hsa-miR-147b-3p*	2.03503	0.00586	*hsa-miR-135a-5p*	−2.74195	0.01066
*hsa-miR-767-5p*	2.00273	0.00252	*hsa-miR-139-5p*	−2.73971	0.00342
*hsa-miR-4521*	1.39937	0.01336	*hsa-miR-181a-3p*	−2.71786	6.92 × 10^−5^
*hsa-miR-147a*	1.04216	0.00636	*hsa-miR-181b-5p*	−2.00047	0.00252
*hsa-miR-92b-5p*	1.03785	0.04323	*hsa-miR-708-5p*	−1.97112	0.03898
			*hsa-miR-181a-5p*	−1.86390	0.00282
			*hsa-miR-184*	−1.71050	0.04126
			*hsa-miR-708-3p*	−1.63216	0.00505

**Table 3:** Exploratory correlation analysis of the expression levels of the top 20 differentially expressed mRNAs with age and aniridia-associated keratopathy (AAK) grade ^1^.

Upregulated							Downregulated						
	Correlation with Age				Correlation with AAK Grade			Correlation with Age				Correlation with AAK Grade	
	Aniridia		Control		Aniridia			Aniridia		Control		Aniridia	
mRNA	r	*p* adj	r	*p* adj	r	*p* adj	mRNA	r	*p* adj	r	*p* adj	r	*p* adj
*OLFM4*	0.356	0.602	−0.510	0.654	0.379	0.603	*KRT3*	0.102	0.883	−0.116	0.956	−0.146	0.824
*S100A7*	0.259	0.738	0.015	0.988	0.597	0.265	*FAT3*	0.097	0.874	0.112	0.939	−0.220	0.828
*BPIFB1*	0.064	0.945	−0.288	>0.999	0.444	0.449	*JCHAIN*	0.188	0.798	−0.633	0.710	−0.016	>0.999
*SULT1E1*	0.580	0.259	0.127	0.948	0.391	0.603	*TRIM71*	−0.003	>0.999	−0.144	0.918	−0.052	>0.999
*CXCL6*	0.471	0.455	−0.327	>0.999	0.682	0.112	*FIBCD1*	0.001	>0.999	0.077	0.906	0.001	>0.999
*SLC5A5*	0.709	0.115	−0.078	0.930	0.557	0.203	*KRT12*	−0.413	0.474	−0.270	0.998	−0.564	0.217
*CCDC190*	0.033	0.986	−0.628	0.378	0.123	0.872	*LRRTM3*	−0.173	0.952	0.407	>0.999	−0.253	0.816
*CYP1A1*	−0.279	0.730	0.103	0.952	0.181	0.854	*NTF3*	−0.160	0.864	−0.257	0.997	−0.307	0.680
*GALNT8*	0.622	0.265	−0.156	0.946	0.221	0.808	*NRN1*	0.487	0.456	0.219	0.821	0.383	0.559
*BPIFB2*	−0.106	0.925	−0.093	0.912	0.206	0.832	*MGARP*	0.009	>0.999	−0.169	0.938	−0.479	0.388
*HAS2*	0.241	0.402	−0.295	>0.999	0.728	0.078	*TMEM100*	0.311	0.714	−0.104	0.936	0.072	>0.999
*LY6D*	0.435	0.482	0.424	>0.999	0.809	**0.026**	*CLC*	−0.415	0.571	−0.302	>0.999	−0.253	0.788
*SOX21*	−0.062	0.927	−0.248	0.920	−0.062	0.980	*KRT27*	−0.401	0.475	−0.253	0.956	0.030	>0.999
*CELA3B*	0.154	0.852	0.034	0.970	0.251	0.811	*TRPM3*	−0.320	0.261	−0.099	0.923	−0.306	0.709
*TSPAN8*	−0.102	0.911	−0.007	0.988	0.109	0.888	*AMPH*	−0.305	0.671	0.244	0.888	−0.314	0.717
*PCDHB12*	0.592	0.284	−0.218	0.791	0.177	0.802	*PID1*	−0.236	0.412	−0.226	0.830	−0.192	0.847
*CXCL3*	0.243	0.761	−0.596	0.366	0.178	0.831	*KRT24*	−0.796	**0.042**	0.156	0.914	−0.574	0.230
*SORCS2*	0.204	0.770	0.285	0.993	0.275	0.750	*BGN*	0.277	0.702	0.235	0.835	0.242	0.802
*PTPRZ1*	0.506	0.446	−0.240	0.860	0.573	0.276	*WSCD2*	0.132	0.912	0.298	>0.999	0.077	0.992
*BMPR1B*	0.460	0.443	0.033	0.990	0.317	0.766	*LSAMP*	−0.414	0.512	0.297	>0.999	0.021	>0.999

^1^ Spearman correlation analyses were performed to assess associations between differentially expressed mRNAs and age as well as AAK grade in aniridia and control samples. Genes are grouped into upregulated and downregulated mRNAs, and correlation coefficients (r) together with false discovery rate (FDR)-adjusted *p*-values (*p* adj) are reported. Significant *p*-values are shown in bold.

**Table 4:** Exploratory correlation analysis of miRNA expression levels with age and aniridia-associated keratopathy (AAK) grade ^1^.

Upregulated							Downregulated						
	Correlation with Age				Correlation with AAK Grade			Correlation with Age				Correlation with AAK Grade	
	Aniridia		Control		Aniridia			Aniridia		Control		Aniridia	
miRNA	r	*p* adj	r	*p* adj	r	*p* adj	miRNA	r	*p* adj	r	*p* adj	r	*p* adj
*hsa-miR-224-5p*	0.583	0.295	−0.297	>0.999	0.756	**0.038**	*hsa-miR-204-5p*	−0.557	0.261	0.033	0.916	−0.594	0.073
*hsa-miR-224-3p*	0.482	0.197	−0.204	0.917	0.735	**0.025**	*hsa-miR-204-3p*	−0.652	0.256	−0.363	>0.999	−0.749	**0.029**
*hsa-miR-452-5p*	0.420	0.286	−0.367	>0.999	0.724	**0.019**	*hsa-miR-138-5p*	−0.398	0.275	−0.196	0.867	−0.502	0.166
*hsa-miR-147b-3p*	−0.159	0.617	−0.073	0.960	−0.028	0.927	*hsa-miR-135a-5p*	−0.347	0.353	−0.081	>0.999	−0.250	0.460
*hsa-miR-767-5p*	−0.296	0.441	0.342	>0.999	0.171	0.621	*hsa-miR-139-5p*	0.290	0.425	−0.446	>0.999	−0.305	0.454
*hsa-miR-4521*	0.493	0.204	0.086	>0.999	0.291	0.421	*hsa-miR-181a-3p*	−0.506	0.212	−0.068	0.917	−0.368	0.371
*hsa-miR-147a*	−0.031	0.918	−0.042	0.941	−0.280	0.419	*hsa-miR-181b-5p*	−0.261	0.434	−0.284	>0.999	−0.668	**0.038**
*hsa-miR-92b-5p*	−0.285	0.406	0.209	0.999	−0.141	0.664	*hsa-miR-708-5p*	−0.519	0.225	−0.213	>0.999	−0.469	0.194
							*hsa-miR-181a-5p*	−0.232	0.471	−0.231	>0.999	−0.647	**0.044**
							*hsa-miR-184*	−0.404	0.288	−0.081	0.994	−0.312	0.477
							*hsa-miR-708-3p*	−0.524	0.270	0.086	>0.999	−0.313	0.425

^1^ Spearman correlation analysis was performed to assess associations between differentially expressed miRNAs and age as well as AAK grade in aniridia and control samples. miRNAs are grouped into upregulated and downregulated categories. Correlation coefficients (r) and false discovery rate (FDR)-adjusted *p*-values (*p* adj) are reported. Significant *p*-values are shown in bold.

**Table 5:** Target genes of dysregulated microRNAs in corneas of patients with congenital aniridia.

miRNA	Target Genes
hsa-miR-204-5p	*AP1S2, BCL2L2, BDNF, SERINC3, ELOVL6, TRPM3, SNAI2, BIRC2, MALAT1, EZR, MEIS1, ITPR1, VIM, CDC42, DVL3, FZD1, MAP1LC3B, RAB40B, SMAD4, SIX1, SERP1, CXCR4, CDH1, TCF4, FOXM1, RAB22A, TXNIP, MEIS2, TMPRSS3, JAK2, RUNX2, SIRT1, NTRK2, USP47, IL11, TGFBR2, CYBB, SOX4, HMX1, MAP2K1, MMP9, UCA1, HMGA2, HOXA10, MX1, IGFBP2, EDEM1, M6PR, THRB, TCF12, FOXC1, CREB5, ALPL, SNAI1, BCL2, SPDEF, SOST, EFNB2, CREB1, ANKRD13A*
hsa-miR-224-5p	*DIO1, KRAS, AP2M1, PTX3, API5, PHLPP2, TPD52, CDC42, GSK3B, PEBP1, SMAD4, TCEAL1, DPYSL2, PAK2, KLK10, TRIB1, RAC1, EDNRA, APLN, MBD2, CXCR4, SERPINF2, CDH1, RASSF8, BCL2, CASP3, HOXD10, MTOR, EYA4, PHLPP1, CASP7*
hsa-miR-224-3p	*FUT4, RB1CC1, ATG5*
hsa-miR-204-3p	*RGS5, ATF2, KHDRBS1, PPM1K*
hsa-miR-138-5p	*ROCK2, SNAI2, BLCAP, H2AFX, RELN, VIM, EZH2, TERT, RMND5A, CD274, AKT1, NFKB1, ADGRA2, SOX9, S100A1, CDH1, KDM5C, BAG1, HIF1A, CYTOR, CASP3, YAP1, BCL11A, EIF4EBP1, SIRT1, MXD1, RHOC, FOSL1, SOX4, MAP3K11, PTK2, RARA, FOXC1, LCN2, FERMT2, SENP1, CCND3, ZEB2, GNAI2, EID1, SUZ12, CCND1, TWIST2*
hsa-miR-452-5p	*DPYSL2, THRB, CDKN1B, LEF1, KRAS, TCF4, BMI1*
hsa-miR-135a-5p	*VLDLR, ROCK2, MYC, APC, CEBPD, NR3C2, BMPR2, EGFR, FOXO1, PPM1E, PTPRD, TXNIP, DAPK2, E2F1, MMP11, KLF8, JAK2, RUNX2, IRS2, SLC6A4, PHLPP2, MTSS1, PTK2, RBAK, HOXA10, HTR1A, SMAD5, SIAH1, BCL2, KLF4, STAT6, ROCK1, ESRRA*
hsa-miR-139-5p	*ROCK2, MMP11, FAM162A, MCL1, NR5A2, SMARCA4, RHOT1, ZHX2, TPD52, WNT1, OIP5, MET, PDE4D, HRAS, NOTCH1, IGF1R, NFKB1, CXCR4, RAP1B, BCL2, ADGRL4, JUN, FOS, ACTC1, PIK3CA*
hsa-miR-181a-3p	*NANOG*
hsa-miR-767-5p	*COL3A1, FBN1, COL4A1, COL10A1, SPARC, SERPINH1, LOX, COL5A2, MMP2, COL4A2, PDGFRB*
hsa-miR-181b-5p	*SIX2, ATM, CBX7, MCL1, PLAG1, LATS2, TCL1A, MAP3K10, XIAP, GATA6, KPNA4, TIMP3, SPP1, PBX3, RASSF1, E2F1, CYLD, KAT2B, HMGB1, VSNL1, MEG3, PTEN, SIRT1, PDCD10, TMED7, BCL2L11, RNF2, NLK, MAP2K1, IGF1R, ELN, CARD10, RAP1B, BCL2, HK2, GRIA2, PDCD4, FOS, CREB1, CDX2, ADCY9, NFIA*
hsa-miR-708-5p	*CNTFR, TMEM88, PARP1, BIRC5, CD44, AKT2, SMAD3, EZH2, MMP2, BMI1, CD274, AKT1, NNAT, EYA3, CASP2, BCL2, KDM1A, ZEB2, IKBKG, CCND1*
hsa-miR-181a-5p	*STAT3, EGR1, ATM, DDIT4, TWIST1, PLAG1, TERT, AHR, PGR, GATA6, RUNX1, BAX, PRKCD, PBX3, MAPK1, CDKN1B, RASSF1, SAMHD1, KLF6, MEG3, BCL2L11, ATG5, NLK, ABCG2, RALA, CTDSPL, PROX1, RAP1B, RGS16, CTNNB1, BCL2, DDX3X, PTPN22, CDX2, E2F5, DUSP5, TUSC3, MCL1, RASSF6, XIAP, HRAS, MTMR3, PRAP1, TCF4, PRKN, GPD1L, TGFBRAP1, DUSP6, WIF1, INPP4B, RGS5, KAT2B, PTPN11, ZNF763, SIRT1, KRAS, NRAS, GPR78, PHLPP2, RNF2, PPP3CA, MAP2K1, TIMP1, HIPK2, NOTCH1, COL16A1, CEBPA, IFNG, CDKN1A, TGFBR1, FOS, PTEN*
hsa-miR-184	*PRKCB, MYC, AGO2, EZR, AKT2, PLPP3, AKT1, SOX7, PKM, INPPL1, PPP1R13L, BCL2, TNFAIP2, NFATC2, BIN3, GAS1, SND1, PDGFB, ZFPM2*
hsa-miR-708-3p	*TEX261, OTUB, N4BP1, MPL*
hsa-miR-147a	*HIF3A, VEGFA, MCM3, PSMA3, NDUFA4, SLC22A3*

**Table 6:** Demographic characteristics, aniridia-associated keratopathy (AAK) Grade (according to Lagali et al. [7]) and mutation type of subjects with congenital aniridia and demographic characteristics of controls.

Subjects with Congenital Aniridia					Healthy Control Subjects		
Donor Number	Gender	Age (Years)	AAK Grade R/L	Mutation Type	Donor Number	Gender	Age (Years)
1	Female	23	1/1	Splicing	1	Male	6
2	Female	16	1/1	Missence	2	Male	2
3	Male	26	2/2	Nonsense	3	Female	16
4	Male	57	4/4	Nonsense	4	Female	19
5	Male	51	2/2	Microdeletion	5	Female	22
6	Male	11	1/1	Nonsense	6	Female	23
7	Female	11	2/2	Nonsense	7	Female	42
8	Male	23	4/2	Frameshift	8	Female	60
9	Female	23	4/4	Splicing	9	Female	49
10	Male	25	2/-	Frameshift	10	Female	49
11	Male	17	0/0	Splicing	11	Male	9
12	Female	50	2/2	Splicing	12	Male	18
13	Female	14	0/0	Splicing	13	Male	35
14	Female	12	2/2	Splicing	14	Male	59

## Data Availability

The original contributions presented in this study are included in the article/Supplementary Material. Further inquiries can be directed to the corresponding author.

## Supplementary Materials

The following supporting information can be downloaded at: https://www.mdpi.com/article/10.3390/ijms27136088/s1.

## Abbreviations

The following abbreviations are used in this manuscript:

AAK	Aniridia-associated keratopathy
AN-LECs	Aniridia limbal epithelial cells
AN-LSCs	Aniridia limbal stromal cells
BP	Biological process
CA	Congenital aniridia
CC	Cellular component
cDNA	Complementary DNA
DEGs	Differentially expressed genes
FC	Fold change
FDR	False discovery rate
GO	Gene Ontology
GUSB	Glucuronidase beta
IC	Impression cytology
KEGG	Kyoto Encyclopedia of Genes and Genomes
MCC	Maximal clique centrality
MF	Molecular function
miRNA	Micro RNA
mRNA	Messenger RNA
PAX6	Paired box gene 6
PPI	Protein–protein interaction
RPMMM	Reads per million mapped miRNAs
SHH	Sonic hedgehog
TBP	TATA-box binding protein
VST	Variance-stabilizing transformation
