# Supplementary material for: Integrated Analysis of mRNA and microRNA Expression in Corneal Impression Cytology Samples from Patients with PAX6-Related Congenital Aniridia
Source: Int J Mol Sci. 2026 Jul 7;27(13):6088. doi: 10.3390/ijms27136088 (PMC13361358; doi:10.3390/ijms27136088)
Supplement: Supplementary file 1 [file ijms-27-06088-s001.zip › Supplementary Tables_20260702.pdf]

Table S1. Predicted binding sites of dysregulated miRNA–mRNA pairs in corneal impression cytology samples from patients with congenital aniridia.

<table>
<tr><th>Gene and miRNA</th><th>Predicted consequential pairing of target region (top) and miRNA (bottom)</th><th>Position</th><th>Site type</th></tr>
<tr><td>LOX
has-miR-767-5p</td><td>5′ ... AUCCUCUAAGCUAAAAUGGUGCU...
| | | | | | |
3′ GUACCAGUCUGUUGGUACCACGU</td><td>3226-3232 of LOX (3′UTR)</td><td>7mer-m8</td></tr>
<tr><td rowspan="2">HIF3A
has-miR-147a</td><td>5′...UGCACUGGUUCUAGACCACACAA...
| | | | | | |
3′ CGUCUUCGUAAAGGUGUGUG</td><td>793-800 of HIF3A (3′UTR)</td><td>8mer</td></tr>
<tr><td>5′...UGAAGACUUCUCCAGCCACACAAG...
| | | | | | |
3′ CGUCUUCGUAAAGGUGUGUG</td><td>3400-3406 of HIF3A (3′UTR)</td><td>7mer-m8</td></tr>
<tr><td>MEIS2
has-miR-204-5p</td><td>5′...CAUGCCCAAUAGUAUAAGGGAAC...
| | | | | |
3′ UCCGUAUCCUACUGUUUCCCUU</td><td>328-334 of MEIS2 (3′UTR)</td><td>7mer-A1</td></tr>
<tr><td rowspan="2">NTRK2
has-miR-204-5p</td><td>5′... UGAUGUGGAUGAAAAAAAGGGAA...
| | | | | | |
3′ UCCGUAUCCUACUGUUUCCCUU</td><td>457-464 of NTRK2 (3′UTR)</td><td>8mer</td></tr>
<tr><td>5′...GCAGAAUCAAUCCCUAAGGGAA...
| | | | | |
3′ UCCGUAUCCUACUGUUUCCCUU</td><td>1446-1452 of NTRK2 (3′UTR)</td><td>7mer-A1</td></tr>
</table>

<table>
<tr><td></td><td>5′...ACAUGAGCCAGAUUGAAAGGGAG...
| | | | | | |
3′ UCCGUAUCCUACUGUUUCCCUU</td><td>1853-1859 of
NTRK2
(3′UTR)</td><td>7mer-m8</td></tr>
<tr><td>HMGA2
has-miR-204-5p</td><td>5′ ...AUGUUCAUUUUUCAAAAAGGGAA...
| | | | | | |
3′ UCCGUAUCCUACUGUUUCCCUU</td><td>665-672 of
HMGA2
(3′UTR)</td><td>8mer</td></tr>
<tr><td>MYC
has-miR-135a-5p</td><td>5′ ...GAGACUGAAAGAUUUAGCCAUAA...
| | | | | |
3′ AGUGUAUCCUUAUUUUUCGGUAU</td><td>125-131 of
MYC (3′UTR)</td><td>7mer-A1</td></tr>
<tr><td rowspan="2">PTPRD
has-miR-135a-5p</td><td>5′ ... AUGGAGUCUCUUCUGAGCCAUAC...
| | | | | |
3′ AGUGUAUCCUUAUUUUUCGGUAU</td><td>63-69 of
PTPRD
(3′UTR)</td><td>7mer-A1</td></tr>
<tr><td>5′ ... CAGAAUUUCUAUAGGAAGCCAUG...
| | | | | | |
3′ AGUGUAUCCUUAUUUUUCGGUAU</td><td>1974-1980 of
PTPRD
(3′UTR)</td><td>7mer-m8</td></tr>
<tr><td>MTSS1
has-miR-135a-5p</td><td>5′...UAAAUGCAUCCGAAU ------AAGCCAUA...
| | | | | | | | | | |
3′ AGUGUAUCCUUAUUUUUCGGUAU</td><td>705-712 of
MTSS1
(3′UTR)</td><td>8mer</td></tr>
<tr><td>KAT2B
has-miR-181b-5p</td><td>5′... UUUUCAGACCAUGAAUGAAUGUU...
| | | | | | | | | |
3′ UGGGUGGCUGUCGUU-- ACUUACAA</td><td>366-372 of
KAT2B
(3′UTR)</td><td>7mer-m8</td></tr>
</table>

<table>
<tr><td>KAT2B
has-miR-181a-5p</td><td>5′... UUUUCAGACCAUGAAUGAAUGUU...
| | | | | | | | | |
3′ UGAGUGGCUGUCGCA-- ACUUACAA</td><td>366-372 of
KAT2B
(3′UTR)</td><td>7mer-m8</td></tr>
<tr><td>TNFAIP2
has-miR-184</td><td>5′... GCCGGCUCUCUUGGGUCCGUCCC...
| | | | | | |
3′ UGGGAAUAGUCAAGAGGCAGGU</td><td>1870-1876 of
TNFAIP2
(3′UTR)</td><td>7mer-m8</td></tr>
</table>

Table S2. Detailed Gene Ontology (GO) enrichment analysis results for differentially expressed mRNAs in corneal impression cytology samples from patients with congenital aniridia.

Category	Term	Genes	Count	List Total	Pop Hits	Pop Total	P value	Benjamini	Fold Enrichment	Bonferroni	FDR	Fisher Exact
Biological process	Cell adhesion	8.61%	59	616	677	19512	4.92E-12	1.40E-08	2.76	1.40E-08	1.39E-08	1.66E-12
Biological process	Inflammatory response	5.99%	41	616	474	19512	1.73E-08	2.47E-05	2.74	4.95E-05	2.46E-05	5.89E-09
Biological process	Chemotaxis	3.07%	21	616	163	19512	2.20E-07	2.09E-04	4.08	6.26E-04	2.07E-04	4.81E-08
Biological process	Lipid metabolic process	7.88%	54	616	847	19512	1.65E-06	1.15E-03	2.02	4.69E-03	1.15E-03	7.73E-07
Biological process	Homophilic cell-cell adhesion	2.92%	20	616	171	19512	2.02E-06	1.15E-03	3.7	5.75E-03	1.15E-03	4.93E-07
Biological process	Nervous system development	6.42%	44	616	649	19512	4.00E-06	1.90E-03	2.15	1.14E-02	1.89E-03	1.74E-06
Biological process	Cell-cell signaling	3.36%	23	616	236	19512	6.15E-06	2.51E-03	3.09	1.74E-02	2.49E-03	1.80E-06
Biological process	Cell-cell adhesion	3.21%	22	616	225	19512	9.67E-06	3.45E-03	3.1	2.72E-02	3.43E-03	2.86E-06
Biological process	Immune response	5.69%	39	616	572	19512	1.36E-05	4.24E-03	2.16	3.82E-02	4.21E-03	5.99E-06
Biological process	Neuron projection development	2.63%	18	616	162	19512	1.48E-05	4.24E-03	3.52	4.15E-02	4.21E-03	3.83E-06

Cellular component	Plasma membrane	43.50%	298	654	5927	20808	2.81E-21	1.36E-18	1.6	1.36E-18	1.31E-18	1.73E-21
Cellular component	Membrane	53.72%	368	654	9038	20808	1.91E-11	4.59E-09	1.3	9.18E-09	4.43E-09	1.45E-11
Cellular component	Extracellular space	16.35%	112	654	1895	20808	7.08E-11	1.14E-08	1.88	3.41E-08	1.10E-08	3.69E-11
Cellular component	Extracellular region	20.73%	142	654	2693	20808	3.33E-10	4.02E-08	1.68	1.61E-07	3.88E-08	1.94E-10
Cellular component	Extracellular matrix	6.57%	45	654	492	20808	4.97E-10	4.79E-08	2.91	2.39E-07	4.62E-08	1.58E-10
Cellular component	Synapse	8.03%	55	654	1001	20808	7.71E-05	6.19E-03	1.75	3.65E-02	5.97E-03	4.21E-05
Cellular component	Neuron projection	3.94%	27	654	372	20808	1.32E-04	9.12E-03	2.31	6.18E-02	8.79E-03	5.32E-05
Cellular component	Receptor complex	2.77%	19	654	216	20808	1.70E-04	1.02E-02	2.8	7.85E-02	9.86E-03	5.57E-05
Cellular component	Cell surface	5.99%	41	654	701	20808	2.15E-04	1.15E-02	1.86	9.85E-02	1.11E-02	1.09E-04
Cellular component	Cell periphery	1.90%	13	654	116	20808	2.90E-04	1.28E-02	3.57	1.30E-01	1.23E-02	7.33E-05
Molecular function	Extracellular matrix structural constituent	2.77%	19	639	127	19272	2.03E-07	1.83E-04	4.51	1.83E-04	1.82E-04	3.93E-08
Molecular function	Calcium ion binding	7.59%	52	639	744	19272	7.79E-07	2.68E-04	2.11	7.04E-04	2.66E-04	3.47E-07

Molecular function	Structural constituent of skin epidermis	1.90%	13	639	63	19272	8.89E-07	2.68E-04	6.22	8.04E-04	2.66E-04	1.18E-07
Molecular function	RAGE receptor binding	0.88%	6	639	12	19272	2.56E-05	5.77E-03	15.08	2.28E-02	5.74E-03	1.01E-06
Molecular function	Cell adhesion molecule binding	2.04%	14	639	134	19272	5.12E-04	8.57E-02	3.15	3.71E-01	8.51E-02	1.48E-04
Molecular function	Monoatomic ion channel activity	2.48%	17	639	189	19272	5.69E-04	8.57E-02	2.71	4.02E-01	8.51E-02	1.93E-04
Molecular function	Extracellular matrix binding	1.02%	7	639	33	19272	6.64E-04	8.57E-02	6.4	4.51E-01	8.51E-02	8.59E-05
Molecular function	Structural molecule activity	2.63%	18	639	214	19272	7.93E-04	8.96E-02	2.54	5.12E-01	8.90E-02	2.91E-04
Molecular function	Transmembrane receptor protein tyrosine phosphatase activity	0.73%	5	639	17	19272	2.01E-03	1.78E-01	8.87	8.37E-01	1.77E-01	1.75E-04
Molecular function	Signaling receptor activity	3.36%	23	639	339	19272	2.14E-03	1.78E-01	2.05	8.56E-01	1.77E-01	9.93E-04

Table S3. Detailed Gene Ontology (GO) enrichment analysis results for target genes regulated by differentially expressed miRNAs in corneal impression cytology samples from patients with congenital aniridia.

Category	Term	Genes	Count	List Total	Pop Hits	Pop Total	P value	Benjamini	Fold Enrichment	Bonferroni	FDR	Fisher Exact
Biological process	Positive regulation of transcription by RNA polymerase II	29.87%	89	296	1243	19512	5.28E-36	1.80E-32	4.72	1.80E-32	1.64E-32	1.06E-36
Biological process	Negative regulation of transcription by RNA polymerase II	24.16%	72	296	1034	19512	7.45E-28	1.27E-24	4.59	2.54E-24	1.15E-24	1.53E-28
Biological process	Apoptotic process	21.14%	63	296	800	19512	4.71E-27	5.36E-24	5.19	1.61E-23	4.87E-24	8.51E-28
Biological process	Positive regulation of gene expression	17.11%	51	296	515	19512	3.46E-26	2.95E-23	6.53	1.18E-22	2.68E-23	4.86E-27
Biological process	Positive regulation of cell population proliferation	17.45%	52	296	542	19512	4.50E-26	3.07E-23	6.32	1.53E-22	2.78E-23	6.54E-27
Biological process	Negative regulation of apoptotic process	16.44%	49	296	543	19512	2.24E-23	1.27E-20	5.95	7.64E-20	1.16E-20	3.48E-24

Biological process	Positive regulation of DNA-templated transcription	19.46%	58	296	791	19512	2.77E-23	1.35E-20	4.83	9.46E-20	1.23E-20	5.40E-24
Biological process	Positive regulation of apoptotic process	12.42%	37	296	333	19512	1.43E-20	6.09E-18	7.32	4.87E-17	5.52E-18	1.76E-21
Biological process	Regulation of transcription by RNA polymerase II	25.84%	77	296	1643	19512	2.54E-19	9.63E-17	3.09	8.67E-16	8.74E-17	7.96E-20
Biological process	Positive regulation of cell migration	11.41%	34	296	300	19512	3.63E-19	1.24E-16	7.47	1.24E-15	1.12E-16	4.39E-20
Cellular component	Chromatin	24.83%	74	293	1159	20808	2.48E-28	1.11E-25	4.53	1.11E-25	9.83E-26	5.20E-29
Cellular component	Nucleoplasm	44.30%	132	293	4091	20808	4.99E-23	1.11E-20	2.29	2.23E-20	9.88E-21	2.14E-23
Cellular component	Transcription regulator complex	11.07%	33	293	251	20808	1.67E-21	2.49E-19	9.34	7.46E-19	2.20E-19	1.58E-22
Cellular component	Nucleus	58.39%	174	293	7054	20808	2.95E-19	3.30E-17	1.75	1.32E-16	2.92E-17	1.67E-19
Cellular component	Cytoplasm	61.07%	182	293	7749	20808	3.68E-18	3.29E-16	1.67	1.65E-15	2.92E-16	2.19E-18
Cellular component	Cytosol	48.32%	144	293	5677	20808	1.79E-15	1.33E-13	1.8	7.94E-13	1.18E-13	9.48E-16

Cellular component	RNA polymerase II transcription regulator complex	6.71%	20	293	135	20808	5.26E-14	3.36E-12	10.52	2.35E-11	2.98E-12	4.35E-15
Cellular component	Protein-containing complex	12.75%	38	293	797	20808	1.76E-10	9.83E-09	3.39	7.87E-08	8.71E-09	4.98E-11
Cellular component	Focal adhesion	7.38%	22	293	438	20808	1.13E-06	5.61E-05	3.57	5.05E-04	4.97E-05	3.01E-07
Cellular component	Perinuclear region of cytoplasm	9.73%	29	293	737	20808	1.95E-06	8.71E-05	2.79	8.70E-04	7.71E-05	6.71E-07
Molecular function	DNA-binding transcription factor activity	18.12%	54	294	739	19272	1.83E-21	9.77E-19	4.79	1.30E-18	8.64E-19	3.60E-22
Molecular function	Protein binding	91.95%	274	294	13741	19272	3.07E-21	9.77E-19	1.31	2.19E-18	8.64E-19	2.34E-21
Molecular function	DNA-binding transcription activator activity, RNA polymerase II-specific	14.43%	43	294	456	19272	4.12E-21	9.77E-19	6.18	2.93E-18	8.64E-19	6.14E-22
Molecular function	RNA polymerase II cis-regulatory region sequence-specific DNA binding	20.81%	62	294	1100	19272	4.62E-19	8.22E-17	3.69	3.29E-16	7.28E-17	1.20E-19

Molecular function	Transcription cis-regulatory region binding	10.74%	32	294	286	19272	8.08E-18	1.15E-15	7.33	5.75E-15	1.02E-15	9.97E-19
Molecular function	DNA-binding transcription factor activity, RNA polymerase II-specific	21.48%	64	294	1247	19272	1.09E-17	1.29E-15	3.36	7.75E-15	1.14E-15	3.12E-18
Molecular function	Sequence-specific DNA binding	13.42%	40	294	514	19272	9.14E-17	9.30E-15	5.1	7.90E-14	8.23E-15	1.68E-17
Molecular function	Protein kinase binding	13.09%	39	294	491	19272	1.22E-16	1.09E-14	5.21	7.90E-14	9.62E-15	2.20E-17
Molecular function	Sequence-specific double-stranded DNA binding	14.77%	44	294	638	19272	1.58E-16	1.25E-14	4.52	7.90E-14	1.10E-14	3.30E-17
Molecular function	Identical protein binding	23.83%	71	294	1824	19272	2.14E-13	1.53E-11	2.55	1.53E-10	1.35E-11	8.15E-14

Table S4. Detailed Kyoto Encyclopedia of Genes and Genomes (KEGG) enrichment analysis results for differentially expressed mRNAs in corneal impression cytology samples from patients with congenital aniridia.

Category	Term	Genes	Count	List Total	Pop Hits	Pop Total	P value	Benjamini	Fold Enrichment	Bonferroni	FDR	Fisher Exact
KEGG Pathway	Staphylococcus aureus infection	1.90%	17	329	102	9496	3.64E-07	1.07E-04	4.81	1.07E-04	1.04E-04	6.52E-08
KEGG Pathway	Cornified envelope formation	3.36%	23	329	217	9496	5.66E-06	8.32E-04	3.06	1.66E-03	8.07E-04	1.68E-06
KEGG Pathway	Cytoskeleton in muscle cells	3.36%	23	329	233	9496	1.75E-05	1.72E-03	2.85	5.13E-03	1.67E-03	5.65E-06
KEGG Pathway	Cytokine-cytokine receptor interaction	3.80%	26	329	298	9496	3.58E-05	2.63E-03	2.52	1.04E-02	2.55E-03	1.31E-05
KEGG Pathway	Viral protein interaction with cytokine and cytokine receptor	1.90%	13	329	100	9496	1.62E-04	9.50E-03	3.75	4.62E-02	9.21E-03	3.83E-05
KEGG Pathway	Rheumatoid arthritis	1.75%	12	329	95	9496	4.13E-04	2.02E-02	3.65	1.14E-01	1.96E-02	1.01E-04
KEGG Pathway	Arrhythmogenic right ventricular cardiomyopathy	1.46%	10	329	86	9496	2.78E-03	1.06E-01	3.36	5.58E-01	1.03E-01	7.43E-04
KEGG Pathway	Amoebiasis	1.61%	11	329	103	9496	2.89E-03	1.06E-01	3.08	5.71E-01	1.03E-01	8.46E-04

KEGG Pathway	ECM-receptor interaction	1.46%	10	329	89	9496	3.51E-03	1.15E-01	3.24	6.43E-01	1.11E-01	9.73E-04
KEGG Pathway	IL-17 signaling pathway	1.46%	10	329	95	9496	5.42E-03	1.59E-01	3.04	7.97E-01	1.55E-01	1.61E-03
KEGG Pathway	Hypertrophic cardiomyopathy	1.46%	10	329	99	9496	7.09E-03	1.89E-01	2.92	8.76E-01	1.84E-01	2.20E-03
KEGG Pathway	Neuroactive ligand-receptor interaction	3.36%	23	329	370	9496	9.16E-03	2.00E-01	1.79	9.33E-01	1.94E-01	4.76E-03
KEGG Pathway	Cell adhesion molecules	1.90%	13	329	160	9496	9.40E-03	2.00E-01	2.35	9.37E-01	1.94E-01	3.68E-03
KEGG Pathway	Taste transduction	1.31%	9	329	86	9496	9.51E-03	2.00E-01	3.02	9.39E-01	1.94E-01	2.84E-03
KEGG Pathway	Chemokine signaling pathway	2.04%	14	329	193	9496	1.63E-02	3.20E-01	2.09	9.92E-01	3.10E-01	7.17E-03
KEGG Pathway	Vascular smooth muscle contraction	1.61%	11	329	134	9496	1.75E-02	3.22E-01	2.37	9.94E-01	3.12E-01	6.76E-03
KEGG Pathway	Linoleic acid metabolism	0.73%	5	329	30	9496	1.89E-02	3.26E-01	4.81	9.96E-01	3.16E-01	3.38E-03
KEGG Pathway	Hematopoietic cell lineage	1.31%	9	329	100	9496	2.21E-02	3.42E-01	2.6	9.99E-01	3.32E-01	7.71E-03
KEGG Pathway	Estrogen signaling pathway	1.61%	11	329	139	9496	2.21E-02	3.42E-01	2.28	9.99E-01	3.32E-01	8.82E-03

KEGG Pathway	Glycerolipid metabolism	1.02%	7	329	65	9496	2.43E-02	3.47E-01	3.11	9.99E-01	3.36E-01	6.98E-03

Table S5. Detailed Kyoto Encyclopedia of Genes and Genomes (KEGG) enrichment analysis results for target genes regulated by differentially expressed miRNAs in corneal impression cytology samples from patients with congenital aniridia.

Category	Term	Genes	Count	List Total	Pop Hits	Pop Total	P value	Benjamini	Fold Enrichment	Bonferroni	FDR	Fisher Exact
KEGG Pathway	Pathways in cancer	24.83%	74	223	533	9496	1.31E-37	3.51E-35	5.91	3.51E-35	1.54E-35	1.96E-38
KEGG Pathway	AGE-RAGE signaling pathway in diabetic complications	10.74%	32	223	101	9496	7.23E-27	9.65E-25	13.49	1.93E-24	4.23E-25	3.80E-28
KEGG Pathway	Colorectal cancer	10.07%	30	223	87	9496	2.34E-26	2.08E-24	14.68	6.25E-24	9.13E-25	1.09E-27
KEGG Pathway	Proteoglycans in cancer	13.42%	40	223	204	9496	3.48E-25	2.33E-23	8.35	9.30E-23	1.02E-23	3.46E-26
KEGG Pathway	MicroRNAs in cancer	16.11%	48	223	320	9496	4.36E-25	2.33E-23	6.39	1.17E-22	1.02E-23	5.97E-26
KEGG Pathway	Gastric cancer	10.74%	32	223	150	9496	3.43E-21	1.37E-19	9.08	9.16E-19	5.99E-20	3.07E-22
KEGG Pathway	Hepatitis B	11.07%	33	223	163	9496	3.95E-21	1.37E-19	8.62	1.06E-18	5.99E-20	3.77E-22
KEGG Pathway	Prostate cancer	9.40%	28	223	106	9496	4.09E-21	1.37E-19	11.25	1.09E-18	5.99E-20	2.78E-22
KEGG Pathway	Human cytomegalovirus infection	12.42%	37	223	227	9496	2.05E-20	6.07E-19	6.94	5.46E-18	2.66E-19	2.54E-21
KEGG Pathway	Human papillomavirus infection	14.43%	43	223	333	9496	7.65E-20	2.04E-18	5.5	2.04E-17	8.95E-19	1.24E-20

KEGG Pathway	Hepatocellular carcinoma	10.74%	32	223	170	9496	1.67E-19	4.06E-18	8.02	4.46E-17	1.78E-18	1.75E-20
KEGG Pathway	EGFR tyrosine kinase inhibitor resistance	8.05%	24	223	80	9496	1.95E-19	4.33E-18	12.77	5.19E-17	1.90E-18	1.11E-20
KEGG Pathway	PI3K-Akt signaling pathway	14.77%	44	223	362	9496	2.69E-19	5.52E-18	5.18	7.17E-17	2.42E-18	4.68E-20
KEGG Pathway	Chemical carcinogenesis - receptor activation	11.74%	35	223	217	9496	3.81E-19	7.27E-18	6.87	1.02E-16	3.19E-18	4.79E-20
KEGG Pathway	Human T-cell leukemia virus 1 infection	11.74%	35	223	224	9496	1.06E-18	1.89E-17	6.65	2.84E-16	8.30E-18	1.39E-19
KEGG Pathway	Kaposi sarcoma-associated herpesvirus infection	11.07%	33	223	196	9496	1.31E-18	2.18E-17	7.17	3.49E-16	9.57E-18	1.56E-19
KEGG Pathway	Pancreatic cancer	7.72%	23	223	77	9496	1.42E-18	2.24E-17	12.72	3.80E-16	9.80E-18	8.19E-20
KEGG Pathway	Focal adhesion	11.07%	33	223	203	9496	3.84E-18	5.69E-17	6.92	1.02E-15	2.49E-17	4.78E-19
KEGG Pathway	Cellular senescence	9.73%	29	223	157	9496	1.96E-17	2.76E-16	7.87	5.24E-15	1.21E-16	2.10E-18
KEGG Pathway	Chronic myeloid leukemia	7.38%	22	223	77	9496	2.57E-17	3.43E-16	12.17	6.85E-15	1.50E-16	1.57E-18
